# Arbuscular mycorrhizal fungi– linked microbial processes in soil nitrogen cycling

**DOI:** 10.1093/femsec/fiag050

**Published:** 2026-05-12

**Authors:** Anukool Vaishnav, Jan Jansa

**Affiliations:** Laboratory of Fungal Biology, Institute of Microbiology, Czech Academy Sciences, Vídeňská 1083, Praha 4, Czech Republic; Laboratory of Fungal Biology, Institute of Microbiology, Czech Academy Sciences, Vídeňská 1083, Praha 4, Czech Republic

**Keywords:** arbuscular mycorrhiza fungi (AMF), hyphosphere microbiome, nitrogen cycling, nitrification and denitrification, nutrient stoichiometry, organic nitrogen mobilization

## Abstract

Arbuscular mycorrhizal fungi (AMF) create an extended plant-soil interface (referred to as mycorrhizal hyphosphere) where specific microbial interactions shape key steps of the nitrogen (N) cycle. Extraradical hyphae of the AMF host diverse microbes that help mineralize organic substrates, regulate ammonium and nitrate transformations, and enhance N retention. These processes allow plants to access both inorganic N and the N released from complex organic compounds. This review synthesizes current evidence for AMF-microbe interactions in relation to mineralization, nitrification, denitrification, and (di)nitrogen fixation. It also highlights unresolved questions, such as when AMF transition from facilitating to competing for N, how they access stabilized organic N pools, and how the carbon: nitrogen: phosphorus (C: N: P) nutrient stoichiometry of the soil environment constrain AMF-mediated N transfer to plants. Furthermore, we discuss how AMF-centered pathways can contribute to more circular N flows in agroecosystems by promoting tighter internal N cycling through microbial immobilization, turnover, and subsequent transfer to plants via AMF hyphae. By integrating spatial, microbial, and stoichiometric perspectives, this review provides a mechanistic framework for understanding AMF-driven N dynamics and their potential role in enhancing N use efficiency in managed and natural systems.

## Teaser

Arbuscular mycorrhizal fungi participate in soil-plant nitrogen (re)cycling, competing with some and being aided by other microbes. Therefore, microbial processes in mycorrhizal hyphosphere make the centerpoint of this review.

## Introduction

**Figure 1:**
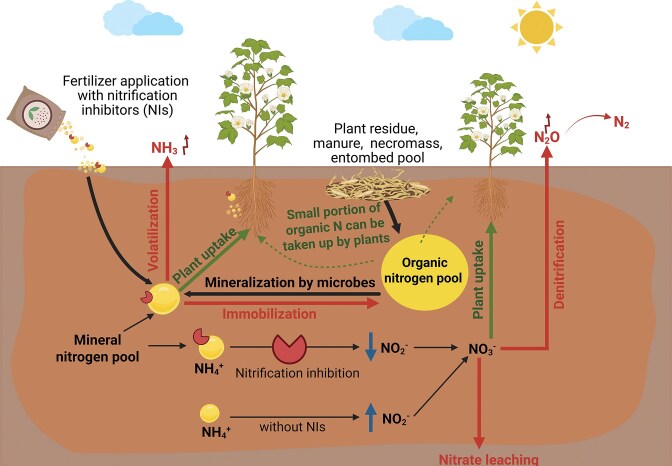
Conceptual overview of major N transformation pathways, N losses, and the influence of (synthetic) nitrification inhibitors in agricultural soils. Key pathways through which N enters, transforms within, and exits agricultural soils. Organic N, which often constitutes majority of total soil N, originates from plant residues, manure, microbial necromass and other detrital pools. Microbial mineralization converts organic N to ammonium (NH₄⁺), which can be immobilized into microbial biomass, taken up by plants (either directly or via the mycorrhizal pathway) or undergo further transformations. A small portion of organic N may be taken up directly by plants in form of amino acids, peptides, and urea. Mineral N, usually representing only a small fraction of total soil N at any given time, is primarily present as NH₄⁺ and nitrate (NO₃⁻). In the absence of biological or (in agroecosystems more common) synthetic nitrification inhibitors (NIs), NH₄⁺ is rapidly oxidized to nitrite (NO₂⁻) and NO₃⁻, increasing susceptibility to nitrate leaching and denitrification N losses. Application of N fertilizers with NIs slows the conversion of NH₄⁺ to NO₂⁻ (the rate limiting step of nitrification), thereby retaining N in ammoniacal form for longer periods of time, and reducing NO₃⁻ accumulation in consequence. Major N loss pathways include ammonia volatilization, NO₃⁻ leaching, and gaseous losses through denitrification (N₂O and N₂ emissions). Plant roots acquire N from both mineral and (to a more limited extent also from the) organic pools. Although NIs can lower NO₃⁻ mediated losses, the potential risks of repeated NI use are often overlooked. These include their leaching into groundwaters, microbial degradation and the emergence of microbial resistance, toxicity to sensitive soil communities and possible entry into the food chain, all of which require further investigation. Created with BioRender.com.

The Green Revolution introduced high-yielding crop varieties and widespread use of synthetic nitrogen (N) fertilizers (Hirsch and Mauchline 2015). This transformation significantly boosted food production, helping to alleviate hunger in many parts of the world. Despite their benefits, the widespread and massive use of synthetic N fertilizers has led to several environmental issues. Reports indicate that about 40–50% of those fertilizers applied to soils are usually lost through leaching or emissions (Zhang et al. 2015). Excess ammonium ions are converted into nitrate through natural nitrification process, which can then leach into groundwater and surrounding water bodies and are thus responsible for eutrophication (Camargo and Alonso 2006). Additionally, nitrate can be converted to nitrous oxide (N_2_O), a potent greenhouse gas (GHG) through denitrification, contributing a major portion of GHG emissions from agriculture (Wrage et al. 2001). These losses not only reduce the fertilizer use efficiency but also contribute to environmental degradation and economic losses for farmers. At present, the global annual demand for synthetic N fertilizer reached above 100 million tonnes and is projected to increase by an additional 200 million tonnes by 2050, underscoring the scale of dependence on these chemicals (FAO 2022, Mirzaee and Nafchi 2025). To reduce N losses associated with synthetic N fertilizers, synthetic nitrification inhibitors (SNIs) were developed to slow the microbial conversion of ammonium to nitrate in the soil (Kim et al. 2012, Meng et al. 2021). These inhibitors can improve retention of N and help lower GHG emissions (Klimczyk et al. 2021, Tufail et al. 2022). However, concerns remain regarding their long-term use, including disruption of non-target microbial communities, potential residue persistence in soil, diminished efficacy over time leading to nitrifier resistance, and variability in effectiveness under different soil pH conditions (Corrochano‐Monsalve et al. 2025) (Fig. 1). This progression, from the initial benefits of agrochemicals to the recognition of their environmental shortcomings and costs underscores the need for a balanced approach that considers both the immediate benefits of increased crop nutrition and yields, and the long-term sustainability of soil ecosystems.

A promising approach is to better utilize the organic N (ON) already present in soils (Knicker 2011, Farzadfar et al. 2021). The ON accounts for more than 90% of total soil N in most agricultural systems, except immediately after inorganic fertilizer application when the fraction of total soil N represented by inorganic forms increases (Kelley and Stevenson 1995, Schulten and Schnitzer 1997, Sun et al. 2025). The ON consists mainly of plant and animal residues, microbial biomass, and humus. A large fraction becomes stabilized as microbial necromass and forms so called “entombed N pool”, protected within aggregates or mineral associations and often referred to as mineral associated organic matter—MAOM (Underwood et al. 2024), which may represent up to half of the soil ON (Liang et al. 2019, Chang et al. 2024). While this stabilization enhances soil carbon (C) and N retention, it also renders the short-term availability to plants. Organic N occurs mainly as amino acids, peptides, proteins, oligo- or polymeric amino sugars such as chitin, and nucleic acids (chiefly DNA), many of which forms are not directly available to plants (Farzadfar et al. 2021). Soil microorganisms convert these compounds into plant-available forms through processes such as desorption, depolymerization and/or deamination, collectively referred to as “mineralization” or “ammonification” (Schimel and Bennett 2004, Kuypers et al. 2018). In unmanaged or low-input ecosystems, plant N supply depends largely on microbial biomass turnover, including cycles of growth, death, and mineralization that release inorganic N (Näsholm et al. 2009). In contrast, intensive agriculture relies heavily on synthetic N fertilizers, often altering microbial communities and reducing their capacity to mineralize ON (Ramirez et al. 2012). Enhancing microbial activity can help unlock stabilized ON, improving plant N supply while avoiding or reducing the environmental impacts of synthetic N fertilizers.

Arbuscular mycorrhizal fungi (AMF) are increasingly recognized for their role in enhancing plant acquisition of N from the ON pool (Hodge and Fitter 2010, Bukovská et al. 2021, Rozmoš et al. 2022). AMF form symbiotic relationship with most terrestrial plants, producing arbuscules or coils inside roots (Dickson 2004). They extend their extraradical hyphae into the soil, which increases the effective nutrient-absorbing surface area of the host plant (Jansa et al. 2019), and create localized zone of direct influence of the hyphae on the surrounding soil, referred to as hyphosphere (Kakouridis et al. 2024). Hyphosphere is the soil zone extending several micrometers to a few millimetres from actively growing AMF hyphae, which is affected by nutrient uptake processes, creating gradients and leading to depletion of diffusion-limited nutrients such as P, and by hyphal exudation and respiration affecting microbial processes in the vicinity of the hyphae. Spatial extent of the hyphosphere will differ according to the perspective (e.g. looking at water, soil biological activity, or P depletion), similarly as in the rhizosphere (Hinsinger et al. 2009). The extraradical hyphae release a range of exudates, including sugars, amino acids, organic acids and other low molecular weight metabolites (Wang et al. 2022). These exudates are thought to provide labile C to hyphosphere microorganisms and influence the chemical and physical properties of the hyphosphere soil. Changes in soil properties may indirectly facilitate N mobilization by modulating microbial activity in the hyphosphere. AMF cannot directly take up complex ON compounds due to their lack of the extracellular enzymes required to depolymerization (Jansa et al. 2019, Faghihinia et al. 2023). Hyphosphere microbes enzymatically depolymerize ON, releasing small organic molecules or ammonium, which can subsequently be taken up by AMF and transferred to the host plant (Vaishnav et al. 2025a, Rozmoš et al. 2022). This relationship is consistent with the AMF priming effect, where C released from AMF hyphae stimulates microbial decomposition of soil organic matter, increasing N mineralization (Zhang et al. 2022). These local interactions thus have significant implications for soil C turnover, nutrient availability in the hyphosphere and ultimately for plant nutrition (Veresoglou et al. 2012, Faghihinia et al. 2023).

Beyond mobilizing ON, AMF alter microbial N cycling processes in the hyphosphere. These interactions can influence nitrification, reduce nitrate leaching, promote complete denitrification, and lower N₂O emissions (Bender et al. 2015, Lyu et al. 2024, Basiru et al. 2025). Strengthening our understanding of plant-AMF interactions, especially in systems receiving organic inputs, could improve N use efficiency, reduce reliance on synthetic N fertilizers, and limit N losses while maintaining ecosystem productivity (Hestrin et al. 2019).

Earlier reviews addressed AMF contributions to N cycling (Veresoglou et al. 2012, Hodge and Storer 2015) and to N uptake from the ON pool (Talbot and Treseder 2010), but these works largely overlooked the role of hyphosphere microorganisms. Recent reviews have acknowledged microbial activity in the hyphosphere (Wang et al. 2022, Faghihinia et al. 2023) but did not examine how AMF-hyphosphere interactions influence specific N transformations pathways. Our previous review summarized early evidence linking AMF, hyphosphere microbes, and ON turnover (Jansa et al. 2019), yet substantial mechanistic advances emerged since then (Vaishnav et al. 2025a, Rozmoš et al. 2022). This mini-review synthesizes the latest progress on: (i) AMF-mediated N acquisition and transfer to plants, (ii) N transformations driven by hyphosphere microbes, and (iii) the combined effects of these processes on soil-plant N cycling. We also identify key knowledge gaps, including whether plant-AMF-associated microbes interactions lead to competition or facilitation with respect to plant N uptake, how AMF access different soil N pools, what are implications of AMF functional diversity, and how these processes could support (more) circular N management in agroecosystems.

## AMF- mediated N uptake and transfer

The AMF are obligate biotrophs that fully depend on plant-derived C for their energy supply and, in return, contribute to plant nutrient delivery, including N. The C supplied by the plant regulates the N uptake via the AMF pathway and its subsequent assimilation in plants, indicating a tight coupling between plant C allocation and fungal nutrient transfer (Fellbaum et al. 2012, Basiru and Hijri 2024). AMF acquire inorganic N [both ammonium (NH₄⁺) and nitrate (NO₃⁻) ions, with no information available for nitrite as yet] directly from soil through specialized membrane transporters located in their extraradical mycelium (Govindarajulu et al 2005, Jin et al 2005).

Variations in AMF traits, such as mycelium development, hyphal morphology and nutrient transporters expression, affect the amount and forms of N acquired and its transfer efficiency to host plants. Trait-related factors like hyphal length density, branching intensity, and spatial proliferation into nutrient-rich patches determine the soil volume explored and the likelihood of encountering mineral or ON sources (Jansa et al. 2019, Kakouridis et al. 2024). Different AMF species allocate resources differently between extraradical and intraradical structures, influencing their nutrient foraging strategies and competition for soil N (Vaishnav et al. 2025b, Lekberg et al. 2024). Finer hyphae and higher branching rates may increase surface-to-volume ratios and nutrient absorption capacity, although this may also raise fungal N demand due to greater biomass turnover. Further, variation in fungal C: N ratios, growth rates, and biomass turnover may further affect fungal N demand and the balance between N retention in the fungal biomass and transfer to plants (Johnson 2010, Fellbaum et al. 2012).

Transporter expression constitutes another critical functional trait. AMF possess high-affinity ammonium transporters (e.g. *GintAMT1*) and nitrate transporters (e.g. *GiNT*), which are expressed in the extraradical mycelium and enable uptake of inorganic N forms from the soil solution (López-Pedrosa et al. 2006, Tian et al. 2010). Ammonium is often preferred for the uptake because its assimilation into amino acids requires less energy than nitrate incorporation into the biomolecules, as the latter necessitates assimilatory reduction to ammonium (Hawkins and George 2001, Xie et al. 2025). Within the fungal hyphae, assimilated N is primarily incorporated into arginine, which serves as a transport and storage molecule. Arginine is later broken down by fungal arginase/urease in intraradical hyphae to release ammonium for transfer into the root cortical cells, a process regulated by plant C supply (Fellbaum et al. 2012).

Beyond inorganic N, genomic and molecular studies indicate that AMF possess transporters for amino acids and small peptides. For example, amino acid permease (e.g. *GmosAAP1*) and oligopeptide transporter (e.g. *RiPTR2*) genes have been identified in *Glomus* (now *Funneliformis*) *mosseae* and *Rhizophagus irregularis*, respectively, and functional tests have shown uptake of dipeptides via such transporters in heterologous (yeast) expression systems (Cappellazzo et al. 2008, Tisserant et al. 2013, Belmondo et al. 2014). Isotopic and fluorescent tracer studies have suggested transfer of amino acids from soil to plants via AMF, although generally a smaller proportion of amino acid-derived N is delivered to the plant relative to inorganic N sources (Hawkins et al. 2000, Whiteside et al. 2012). Direct uptake of other compounds derived from soil ON (e.g. aminosugars) by AMF has not yet been demonstrated convincingly and remains a key unresolved question.

Given the uncertainty about direct AMF uptake of ON, the N acquisition via AMF pathway must be understood within the broader hyphosphere context, where AMF, microbes, and roots interact around shared N pools. The following section examines how these hyphosphere microbial communities regulate N transformations at the soil-AMF interface.

## The hyphosphere as a microbial hotspot for N transformations

The hyphosphere of AMF exhibits local gradients in nutrients, moisture, oxygen and pH. These gradients often arise from microbial activity, exudation, and nutrient uptake by AMF and associated microorganisms (Zhang et al. 2022). This area is characterized by spatial patchiness and dynamic changes as hyphae grow, senesce, and respond to resource heterogeneity. In the hyphosphere, AMF hyphal surfaces serve not only as physical structures for microbial colonization but also facilitate biofilm formation (Vieira et al. 2025). The microbial communities, shaped by the presence of AMF hyphae (Zhang et al. 2022, Duan et al. 2024) contribute to nutrient mobilization, support AMF growth, and may protect the hyphae from grazers through production of bioactive compounds and through biofilm formation (Taktek et al. 2015, Jansa and Hodge 2021). Several studies have shown that AMF hyphae recruit specific bacterial taxa, resulting in communities that differ from bulk soil. Reported taxa include, for example, Betaproteobacteriales, Myxococcales, Fibrobacterales, Cytophagales, Chloroflexales and Cellvibrionales (Emmett et al. 2021, Duan et al. 2024). A core hyphosphere microbiome dominated by Alphaproteobacteria, Actinobacteria and Gammaproteobacteria has been observed across different climate zones and was linked to organic P mineralization (Wang et al. 2023). The composition of these communities can shift depending on AMF species identity, indicating that fungal genotype influences microbial recruitment (Zhou et al. 2023, Lahrach et al. 2024). Microbial guilds associated with N cycling are also consistently detected in the hyphosphere. The following sub-sections synthesize current evidence for specific N cycling pathways in the AMF hyphosphere and examine how microbial activity modulates N fluxes through mineralization, nitrification, and related processes.

### AMF- microbe contributions to N mineralization and dissimilatory nitrate ammonification

In the hyphosphere, microbial degradation of proteins, peptides, amino sugars and other organic substrates releases ammonium (NH₄⁺) that AMF can take up via their extraradical hyphae. Hyphal exudation of labile C accelerates the turnover of organic substrates, resulting in so called priming effect, as well as in nitrate ammonification (Paterson et al. 2016, Zhao et al. 2025). Microbial grazing further enhances this process by releasing N from microbial biomass (the microbial loop), increasing ammonium available to AMF (Bonkowski 2004, Ekelund et al. 2009). These multi-kingdom interactions substantially contribute to plant N acquisition at ecosystem scales, with global estimates suggesting that AMF-saprotroph interactions mobilize approximately 70 Tg N year⁻¹ (Hestrin et al. 2019). Experimental studies show that mycorrhizal plants acquire more N from the ON than non-mycorrhizal plants. For instance, in *Plantago lanceolata*, colonization by *Glomus* (now *Rhizophagus*) *intraradices* enabled plant N uptake from a ^15^N‐labelled organic patch accessed by extraradical fungal hyphae only (Leigh et al. 2009, Thirkell et al. 2016). *Andropogon gerardii* colonized with AMF obtained up to 20 times more ¹⁵N from organic sources and produced six times more biomass than non-mycorrhizal controls (Bukovská et al. 2018). Dudáš et al. (2022) similarly reported that more than 10% of litter-derived ¹⁵N was transferred to plants via AMF within 42 days, demonstrating rapid coupling between ON decomposition and plant N nutrition. In addition, recent work has begun to identify the microbial guilds responsible for ON mineralization in the hyphosphere. Chitinolytic bacteria such as *Paenibacillus* spp. mobilize N from chitin which is then transferred via the AMF pathway to plants (Rozmoš et al. 2022). The amoebozoan protist *Polysphondylium pallidum* increases protein-N mineralization and enhances AMF-mediated N uptake, while AMF hyphae stimulate protist proliferation in protein-rich microsites (Vaishnav et al. 2025a). Other work indicates that AMF may actively recruit specific microbial guilds along extraradical hyphae, extending their foraging range into patchy N sources (such as DNA, clover biomass, or chitin) located beyond the rhizosphere, including genera such as *Pseudoarthrobacter, Nocardioides*, and *Paraparentocirrus* (Vaishnav et al. 2025b).

The efficiency of multi-kingdom interactions in the hyphosphere may also influence access to N from entombed N pools. Hyphal activity can stimulate microbial priming, production of organic acid and extracellular enzyme secretions by the associative microbial communities, which then facilitate desorption and cleavage of the ON compounds (Hodge and Fitter 2010, Bukovská et al. 2018). Through their effects on soil aggregation and microaggregate dynamics, AMF may also modify the physical protection of organic matter, potentially exposing occluded organic matter to microbial attack (Rillig et al. 2015, Lehmann et al. 2020). AMF have been shown to assimilate N derived from microbial necromass when decomposers are present (Chowdhury et al. 2022), and AMF necromass itself can shift hyphosphere microbial communities in ways that benefit the host plant (Jansa et al. 2020). These observations suggest that AMF and their hyphosphere microbiome may influence N release not only from readily degradable substrates but also from stabilized pools, though targeted studies are still needed.

Beyond mineralization, AMF hyphae also influence nitrate ammonification. In a recent study, *Paenibacillus* sp., showing higher abundance in the hyphosphere than in bulk soil, expressed *nirBD* genes that reduced nitrite to ammonium, producing more ¹⁵NH₄⁺ from ¹⁵NO₃⁻ in the hyphosphere than in bulk soil (Zhao et al. 2025). Because AMF preferentially assimilate ammonium, this pathway may be ecologically important. These results also point to a possible role for dissimilatory nitrate reduction to ammonium (DNRA) in the hyphosphere, although evidence remains mixed. AMF inoculation has previously been reported to promote ammonium retention and increase plant N uptake alongside shifts in *nirK/nirS* and *nrfA/nrfH* gene abundances suggestive of DNRA activity (Xing et al. 2025). In contrast, AMF systematically reduced the abundance of DNRA genes such as *napA* and *nrfA* across 50 agricultural soils (Sun et al. 2025), alongside elevated levels of ammonium and reduced nitrate levels in the hyphosphere at the same time. DNRA activity can be evaluated using isotope tracing combined with functional gene analysis (e.g. *nrfA* expression). In addition, ^15^N-labeled nitrate addition allows partitioning of nitrate reduction pathways by tracking the production of ^15^NH_4_⁺ versus gaseous ^15^ N products, thereby distinguishing DNRA from denitrification (Silver et al. 2001, Rütting et al. 2011).

Furthermore, by retaining N in reduced (i.e. less mobile) form, AMF-associated microbes can help limit N losses. In an isotope-labelling study, AMF inoculation reduced N losses by up to 50% during mineralization of chitin and clover biomass and increased N transfer to host plants compared with non-mycorrhizal treatments (Vaishnav et al. 2025b).

### AMF influence on nitrification and ammonium retention

The AMF hyphosphere hosts microbial groups involved in nitrification, including ammonia oxidizing archaea (AOA), ammonia oxidizing bacteria (AOB), nitrite oxidizing bacteria, and complete ammonia oxidizing (comammox) *Nitrospira* (Jansa et al. 2019, Zhu et al. 2022). Although AOA and AOB are autotrophic and less responsive to AMF-derived C than heterotrophs, AMF can still influence their activity by altering N availability and local soil conditions (Veresoglou et al. 2011). One of the reason is that AMF suppress nitrification primarily through competition for ammonium and by promoting ammonium immobilization in microbial biomass (Bukovská et al. 2018, Dudáš et al. 2022, Basiru et al. 2025). The consequences are usually most pronounced for AOB, which are weaker competitors for ammonium than the AMF or AOA (Chen et al. 2013). Consistent with this, AMF often alter ammonia oxidizing community structure under both low and high N fertility conditions (Veresoglou et al. 2011, 2019). AOB play a dominant role in nitrification in agricultural soils, and thus AMF effects often manifest as suppression of AOB-driven nitrification (Chen et al. 2013).

Microcosm studies provide strong support for AOB suppression by AMF. *Rhizophagus irregularis* LPA9 reduced AOB abundance while leaving AOA unchanged in agricultural soils, whereas in artificial substrates both groups were suppressed (Sun et al. 2023, 2024). In a recent study using 50 different agricultural soils, AMF consistently reduced AOB and comammox *Nitrospira*, while AOA remained largely unaffected. AMF-inoculated soils consistently maintained higher NH₄⁺ concentrations than non-mycorrhizal controls, reflecting more effective mineralization, reduced nitrification or increased microbial N retention (Sun et al. 2025). AOB suppression often correlates more strongly with soil pH than with ammonium availability, indicating that AMF effects extend beyond simple resource competition (Jiang et al. 2015, Zhong et al. 2023). In some cases, the extent of AMF suppression of AOB is comparable to synthetic nitrification inhibitors such as dicyandiamide or nitrapyrin (Dudáš et al. 2022), raising the possibility that AMF or AMF-associated microbes may produce biological nitrification inhibitors that move along (or within) the hyphae.

In contrast, under certain environmental conditions- such as high ammonium availability, adequate soil aeration, and pH levels that favour ammonia oxidation, AMF can improve soil structure and promote organic matter turnover, which, in turn, may indirectly support the growth of nitrifiers (Bukovská et al. 2016, Morrison et al. 2017, Teutscherova et al. 2019). AMF hyphal proliferation in nutrient-rich patches has been associated with increased abundance of *Nitrosospira* spp. and an *Acanthamoeba* endosymbiont (Bukovská et al. 2016). Stable isotope probing of C supplied via AMF showed its assimilation by Solibacterales, Sphingobacteriales, Myxococcales and the AOA belonging to Nitrososphaerales (Kakouridis et al. 2024). This may indicate either enhanced C cross-feeding within the microbial communities (mostly relevant for the heterotrophs or mixotrophs) or enhanced ^13^CO_2_ (released by AMF respiration) assimilation within the immediate vicinity of the hyphae by (supposedly) autotrophic AOA. Reduced root exudation following AMF colonization may also shift competitive interactions in favour of nitrifiers by lowering C available to heterotrophic microbes (Jones et al. 2004).

Overall, AMF driven shifts in ammonia oxidizers are thus likely caused by yet poorly understood multisided ecological interactions, including resource competition, modified soil chemistry, and induced changes in other microbial guilds. Clarifying when AMF suppress or stimulate nitrification will require studies that explicitly incorporate soil pH, moisture, texture, and microfaunal interactions. Understanding these dynamics will be the key for leveraging AMF to improve soil N retention and plant N uptake (Basiru et al. 2025).

### AMF regulation of denitrification and gaseous N losses

Because nitrification supplies nitrate for downstream reactions including N losses, understanding how AMF and their hyphosphere microbiome influence denitrification is essential for evaluating soil N retention and gaseous N emissions. The hyphosphere can modulate denitrification through both direct and indirect mechanisms. Directly, AMF affect denitrification by altering nitrate availability and supplying C that serves as an electron donor for denitrifiers (Basiru et al. 2025). Indirectly, AMF influence soil aggregation, aeration, moisture distribution, and pH, all of which shape soil denitrification potential (Okiobe et al. 2022). Despite these mixed pathways, many studies report lower N₂O emissions in AMF-inoculated soils, suggesting that AMF-driven changes in substrate supply and microbial composition often dominate over the other factors.

AMF regulate denitrification by modifying the abundance and community composition of functional guilds that mediate the stepwise reduction of nitrate to gaseous N products. AMF inoculation commonly reduces the relative abundance of N₂O-producing denitrifiers (carrying genes *nirK* or *nirS*) while increasing microbes carrying the N₂O-reducing *nosZ* gene, thereby shifting gaseous end-products from N_2_O toward N₂ (Gui et al. 2021, Li et al. 2022). For instance, *Funneliformis mosseae* suppressed N₂O-producing taxa such as Sphingomonadales and Rhizobiales (He et al. 2023) and increased *nosZ*-I denitrifiers including *Pseudomonas, Achromobacter*, and *Sinorhizobium*, with positive correlations of their abundance to the AMF hyphal length density (Li et al. 2023). Similarly, *Rhizophagus aggregatus* hyphae extending from maize roots into adjacent soybean residues reduced N₂O emissions by 20–61%, accompanied by enrichment of *nosZ*-type denitrifiers (Zhao et al. 2021). AMF hyphae may also act as dispersal networks that redistribute denitrifiers toward microsites enriched in nitrate and C (Jansa and Hodge 2021, Vieira et al. 2025).

### AMF effects on diazotrophs and biological (di)nitrogen fixation (BNF)

The BNF in soils is typically driven by rhizobia-legume symbioses and free-living or associative diazotrophs. Although AMF do not fix atmospheric dinitrogen alone, growing evidence shows that they indirectly influence diazotroph abundance, composition, and activity in the hyphosphere. By altering the physicochemical environment around their extraradical hyphae, AMF create conditions that support N_2_ fixation such as locally lowering O_2_ concentration and providing energy rich compounds through their exudates (Jansa and Hodge 2021). Low-oxygen microsites in the hyphosphere favour nitrogenase activity, which is inhibited by oxygen in many diazotrophs, including *Azotobacter* and *Pseudomonas* (Kuypers et al. 2018). Through these effects, AMF relieve two key constraints on BNF: The C (energy) supply and oxygen sensitivity.

AMF also influence BNF through nutrient-mediated pathways. The N_2_ fixation generally has a high phosphorus (P) demand, and AMF colonization often enhances plant P nutrition (Puppi et al. 1994, Smith and Read 2010). In legumes, improved P supply through AMF increases nodule activity and nitrogenase efficiency, even when plant C has to be shared between AMF and rhizobia (Püschel et al. 2017). Under low N conditions, AMF may therefore improve symbiotic diazotroph performance indirectly by improving P availability and mitigating resource competition between the symbionts (Wang et al. 2021).

Functional evidence confirms that AMF shape diazotroph communities. Hyphosphere-resolved sequencing and *nifH* analyses show enrichment of diazotrophic taxa in AMF-inoculated soils across agricultural and natural ecosystems (Yu et al. 2021, Zhou et al. 2023, Wang et al. 2023). Gou et al. (2024) demonstrated that AMF inoculation increased *nifH* abundance while reducing nitrification-related genes, resulting in greater plant N uptake and lower N loss. These patterns indicate that AMF can shift N cycling toward retention by favoring fixation over nitrification. In addition, previous evidence demonstrated that AMF may harbor intracellular bacteria with genomic potential for dinitrogen fixation. *Burkholderia* endobacteria isolated from AMF structures (Minerdi et al. 2001) suggest that AMF could provide a niche for N₂-fixing bacteria, although direct confirmation of N₂ fixation within AMF hyphae remains lacking to date. Additionally, AMF hyphae can transport *Rhizobium* spp. to legume roots, increasing nodulation and subsequent symbiotic BNF (He et al. 2024).

Collectively, these findings point to AMF as ecosystem engineers that influence mineralization, nitrification, denitrification, and dinitrogen fixation within the hyphosphere. These interactions promote N retention, reduce losses, and enhance plant N acquisition. A clearer understanding will require spatially resolved analyses of gene expression across N cycle pathways, combined with process-based measurements and isotope tracing that distinguish rhizosphere from hyphosphere activities. The next section outlines unresolved issues that limit a complete understanding of AMF-hyphosphere microbial interactions and their potential for improving fertilizer N use efficiency.

## Outstanding questions in AMF-DRIVEN N cycling

### When do AMF shift from N facilitators to competitors?

Plant N acquisition in terrestrial ecosystems reflects a dynamic interplay among plants themselves, AMF, and diverse soil microbes both in the rhizosphere and AMF hyphosphere. Whether AMF function as N facilitators or competitors with their host plants depends on N availability, plant mycorrhizal dependence, and the metabolic demands of surrounding microbial communities (Savolainen and Kytöviita 2022, L’Espérance et al. 2024). When inorganic N is abundant, competition intensifies among plants, AMF, and heterotrophic microbes that depend on similar N pools (Faghihinia et al. 2024, Holz et al. 2025). In contrast, where (structurally heterogeneous and slowly releasing) ON dominates, competition tends to be weaker unless N limitation becomes severe. Under strong N depletion, microbial immobilization may exceed mineralization, limiting N available to both AMF and plants (Basiru et al. 2025). The balance between these outcomes shifts with environmental conditions and plant C allocation, highlighting the need to identify thresholds that determine when AMF switch roles (Lekberg et al. 2024).

Experimental studies show that AMF colonization can reduce plant N uptake under N-limiting conditions, when fungal immobilization outweighs transfer to the host. Püschel et al. (2016) demonstrated direct competition between *Andropogon gerardii* and *Rhizophagus irregularis* under low N supply, and similar reductions in plant N uptake have been reported for maize and other species (Hawkins and George 2001, Toussaint et al. 2004, Reynolds et al. 2005, Wang et al. 2018). Conversely, in systems where ON dominates, AMF often act as facilitators because hyphosphere microbes gradually mineralize complex substrates and continuously supply ammonium to fungal hyphae (see section “AMF-microbe contributions to N mineralization…” for details). The strength of this facilitation depends on substrate availability, microbial demand, and plant C allocation, above all.

A further dimension is the coupled regulation of N, P, and C fluxes. Plant C allocation strongly controls AMF functioning and thus both N and P uptake via the AMF pathway (Hammer et al. 2011). When mineral nutrient supply from the soil is imbalanced, C investment in AMF may shift between P foraging and maintaining N acquisition pathways (Blanke et al. 2005, Hammer et al. 2011). Under P limitation, plants often increase C supply to AMF to enhance P uptake, but this investment can constrain host N assimilation when mineral N is also scarce (Li et al. 2019, Bicharanloo et al. 2020). These dynamics highlight that AMF-mediated nutrient uptake is governed by stoichiometric relationships, rather than responses to individual nutrient pools (Johnson 2010). Yet, current ecosystem and crop models rarely integrate such coupled element cycles. For the above reasons, incorporating an N-P-C stoichiometric framework seems essential to predict when AMF shift from facilitating to competing with plants and to forecast nutrient-use efficiency under climate- and other global changes-driven shifts in C availability and nutrient limitation.

### Which N pools do AMF preferentially exploit?

Although AMF are widely recognized for their role in P acquisition from soil, their nutrient foraging strategies are also relevant for N cycling. Evidence suggests that AMF preferentially exploit readily available P pools, specifically inorganic orthophosphate (Pi), before accessing more complex or spatially distant resources (Etesami and Jeong 2021). When Pi is scarce or tightly sorbed to mineral surfaces, AMF can help mobilize less available nutrient pools by altering rhizosphere chemistry and recruiting phosphate-solubilizing bacteria (PSB). The PSB can enhance dissolution of inorganic P minerals and (together with lytic enzyme producers) mineralization of organic P (Zhang et al. 2014, Andrino et al. 2021). This hierarchical approach to nutrient acquisition highlights the functional plasticity of AMF in heterogeneous soils. However, such hierarchy for exploration of different N pools remains largely unresolved.

**Figure 2:**
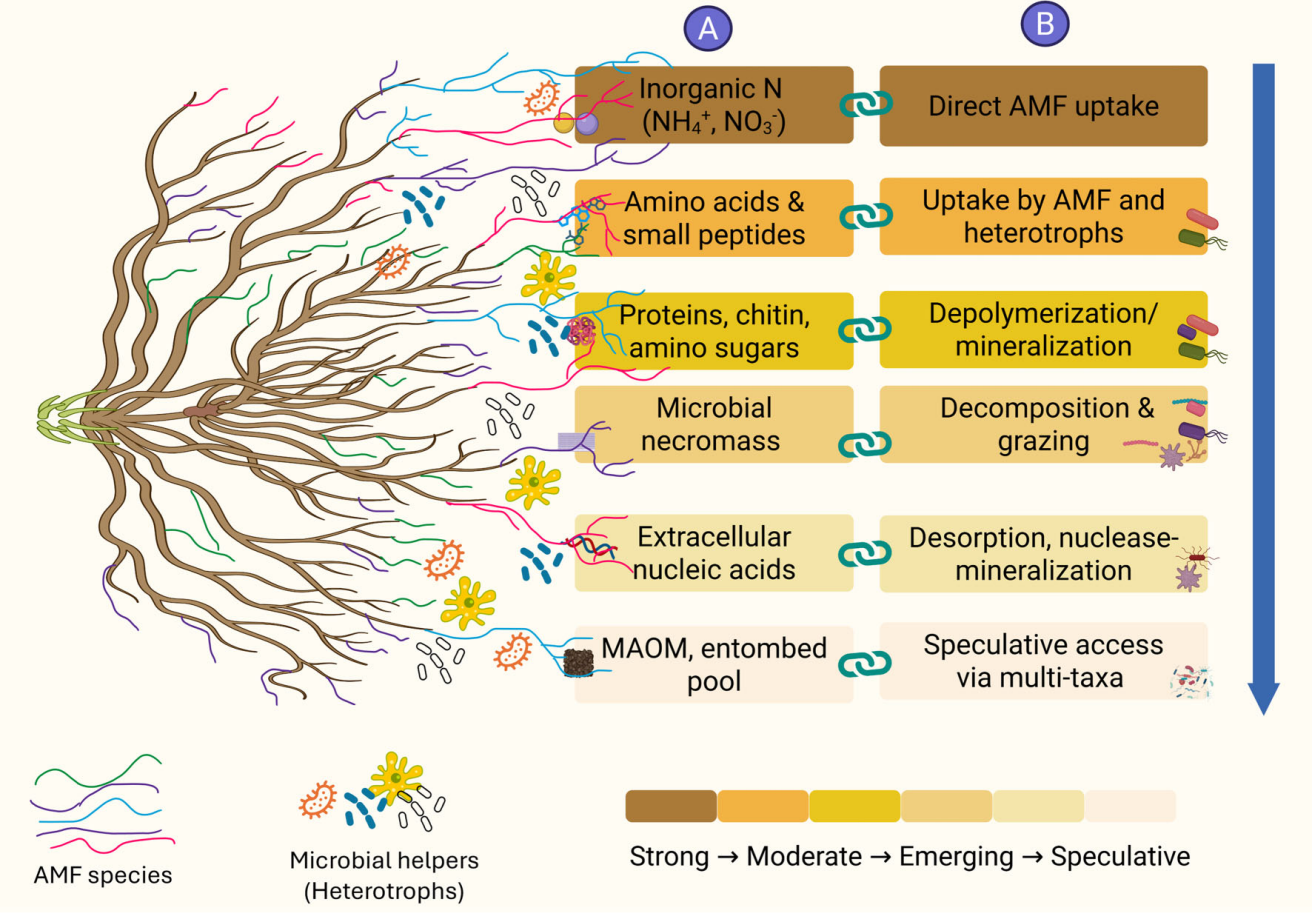
Hierarchical pathways of N foraging by AMF and their microbial partners. Summary of current understanding of the range of N sources accessible to AMF and the hyphosphere microbiome, arranged along a gradient from well-established to speculative pathways. The left panel illustrates the extraradical hyphal network composed of multiple AMF species (co-existing within a single host plant root system), with different hyphae interacting with diverse microbial “helper” guilds. The different foraging behavior of AMF species demonstrates functional complementarity among them (Vaishnav et al. 2025b, Jansa et al. 2008). Panel A depicts the major classes of N substrates present in soil: Inorganic N (such as ammonium, NH₄⁺, and nitrate, NO₃⁻), low-molecular-weight and mostly soluble organic compounds (amino acids and small peptides, urea, mono- or oligomeric amino sugars), polymeric substrates (proteins, chitin and other amino-sugar polymers, DNA and other nucleic acids), microbial necromass (cell wall fragments and cytoplasmic residues), and highly protected entombed N pools such as that contained in the mineral-associated organic matter (MAOM). The shading scale indicates the strength of evidence supporting AMF access or contribution to each substrate class: strong (inorganic N), moderate (amino acids and small peptides), emerging (polymeric N and necromass), and speculative (entombed pools). Panel B summarizes the mechanistic routes through which AMF and hyphosphere microbes access these resources. Inorganic N can be taken up directly by AMF. Amino acids and small peptides represent substrates for shared uptake between AMF and heterotrophs. Polymeric N compounds require microbial desorption, depolymerization and (often also) deamination prior to transfer through AMF hyphae. Microbial necromass is mobilized through saprotrophic decomposition and microbial grazing. Extracellular DNA and other nucleic acids may be mineralized by extracellular nuclease-producing microbes after desorption from soil minerals. Access to entombed N likely depends on multi-taxa consortia capable of disrupting chemically or physically protected matrix structures. Together, the diagram highlights the hierarchical and cooperative nature of AMF-mediated N foraging, emphasizing the increasing role of associated microbial taxa as substrate complexity and/or recalcitrance increases. Created with BioRender.com.

AMF directly use mineral forms of N when available, but their access to organic and particularly the macromolecular N depends on processes occurring in the hyphosphere. As mineral N becomes scarce, AMF increasingly rely on N released through microbial transformations, yet it is not known whether AMF consistently favour particular ON pools over others (Hodge and Storer 2015, Jansa et al. 2019). A major uncertainty is the extent to which AMF can benefit from N held in complex or entombed pools, such as microbial necromass, amino sugars, peptides, or extracellular nucleic acids. Initial evidence suggests that AMF differ in their ability to explore or proliferate in patches containing DNA as soil organic amendment, but the relative contribution of these pools to overall AMF-mediated plant N uptake remains poorly quantified (Vaishnav et al. 2025b, Bukovská et al. 2016). It is also unclear whether AMF exhibit species-specific preferences for particular N forms or whether preferences emerge primarily from the composition of associated microbial communities. AMF species vary markedly in their hyphal traits, nutrient transporters and foraging behavior (see section “AMF- mediated N uptake and transfer” above for details), which can influence soil exploration capacity and fungal N demand (Hart and Reader 2002, Powell et al. 2009). At the same time, the composition of hyphosphere microbial communities has been shown to vary among AMF species (Lahrach et al. 2024), suggesting that fungal identity may indirectly shape their efficacy to access ON pools by recruiting specific microbial partners involved in mineralization, DNRA, or amino acid turnover (Nuccio et al. 2013). These findings raise the possibility that species-level differences in N pool utilization arise from both direct fungal physiological traits and indirect microbially mediated processes. A model framework highlights how substrate complexity governs whether AMF rely on direct uptake or microbially mediated pathways (Fig. 2).

Resolving these questions requires approaches that can explicitly link AMF foraging behavior with N transformation pathways. This includes spatially resolved isotope tracing to separate AMF access to inorganic versus organic N pools, analyses that identify which microbial guilds enable depolymerization and subsequent deamination of complex organic molecules, and experiments that incorporate realistic AMF community mixtures rather than single isolates. Understanding how AMF navigate inorganic, soluble organic, and macromolecular N pools will be essential for predicting their contributions to N retention and use under nutrient limitation, climate stress, and soil degradation.

### Why do AMF exhibit high functional diversity despite limited species richness?

AMF exhibit relatively low taxonomic richness compared to many other soil microbial groups, yet they often demonstrate significant functional diversity, particularly in nutrient acquisition (Koch et al. 2004, Lee et al. 2013). This presents an important research gap in mycorrhizal ecology that highlights the need for a greater attention to the functional complementarity AMF towards N foraging. One reason for the extensive genetic and phenotypic variability within AMF species is that their spores contain multiple nuclei, which can also be genetically distinct (Kokkoris et al. 2020). This characteristic may contribute to high levels of intra-organismal and intra-specific diversity (Sanders et al. 1995, Kuhn et al. 2001). The genetic composition of these nuclei influences variations in physiological traits related to nutrient acquisition and exchange with host plants (Lee et al. 2013). In addition, such genetic makeup may be responsible for notable differences in hyphal growth patterns, transporter expression, and nutrient transfer efficiency, resulting in significant functional differences without the necessity for high species richness.

Moreover, the ecological interactions of AMF can create functional diversity within species. Nowadays, the hyphosphere microbiome is considered a secondary genome of AMF because of its close association with their functioning (Zhang et al. 2022, Wang et al. 2024). This suggests that the contribution of AMF to plant N acquisition might depend partly on species-specific core hyphosphere microbial guilds (Pan and Cai 2026). Additionally, factors like host plant identity, environmental conditions, and soil nutrient availability can further influence the functioning of AMF, enabling a limited number of AMF taxa to express a wide range of functional strategies across different ecosystems (Xu et al. 2017, Fang et al. 2023). For instance, a study demonstrated functional complementarity in foraging for various ON sources among five AMF genera. In this study, *Entrophospora, Acaulospora*, and *Gigaspora* restricted their proliferation to the rhizosphere, whereas *Rhizophagus* and *Funneliformis* explored nutrient patches outside the roots. *Funneliformis* exhibited a preference for DNA and chitin patches, while *Rhizophagus* preferred clover patches (Vaishnav et al. 2025b). *Rhizophagus* also foraged in nutrient-poor patches, such as those enriched with cellulose and starch. These findings suggest a functional complementarity among AMF species in nutrient foraging behaviors. However, it remains unclear whether this functional complementarity arises from genetic variation within AMF species, host filtering responses, or differences in mycelial architecture. Addressing this question will be crucial for predicting when and where the identity of AMF will significantly impact plant N acquisition and soil N partitioning.

### Can AMF support circular N management?

AMF and their hyphosphere microbiomes have the potential to contribute to more circular N flows by improving soil N retention and promoting internal N recycling within soil-plant systems. At the ecosystem scale, AMF presence is associated with lower nitrate leaching and reduced N₂O emissions, patterns consistent with tighter interception of available N by plants and microbes in mycorrhizal systems (Storer et al. 2018, Lyu et al. 2024). AMF-linked processes also support internal recycling by channeling N released from decomposing organic matter into plant uptake pathways (Bunn et al. 2019), while interactions with diazotrophs may reinforce N inputs under low-N conditions. However, translating these biological mechanisms into a circular N management framework requires careful alignment between N sources, synchrony between supply and demand, soil conditions, and AMF activity. AMF-driven recycling cannot (at least not fully and on a long run) replace N exported in harvested biomass, so recycled inputs such as residues, compost, manure, or digestate must be returned to the system. Their effectiveness will depend on how well they coincide with periods of active hyphal growth and turnover, when microbial mineralization and AMF-mediated N capture are most responsive. In addition, AMF effects on N retention vary widely with soil texture, fertility, climate, crop traits, and AMF community composition. This context dependence makes long-term, multi-site field trials and mechanistic N-budgeting approaches essential for evaluating whether AMF-centered strategies can reliably reduce synthetic N fertilizer requirements without compromising yields (Wang et al. 2025a,b).

**Figure 3:**
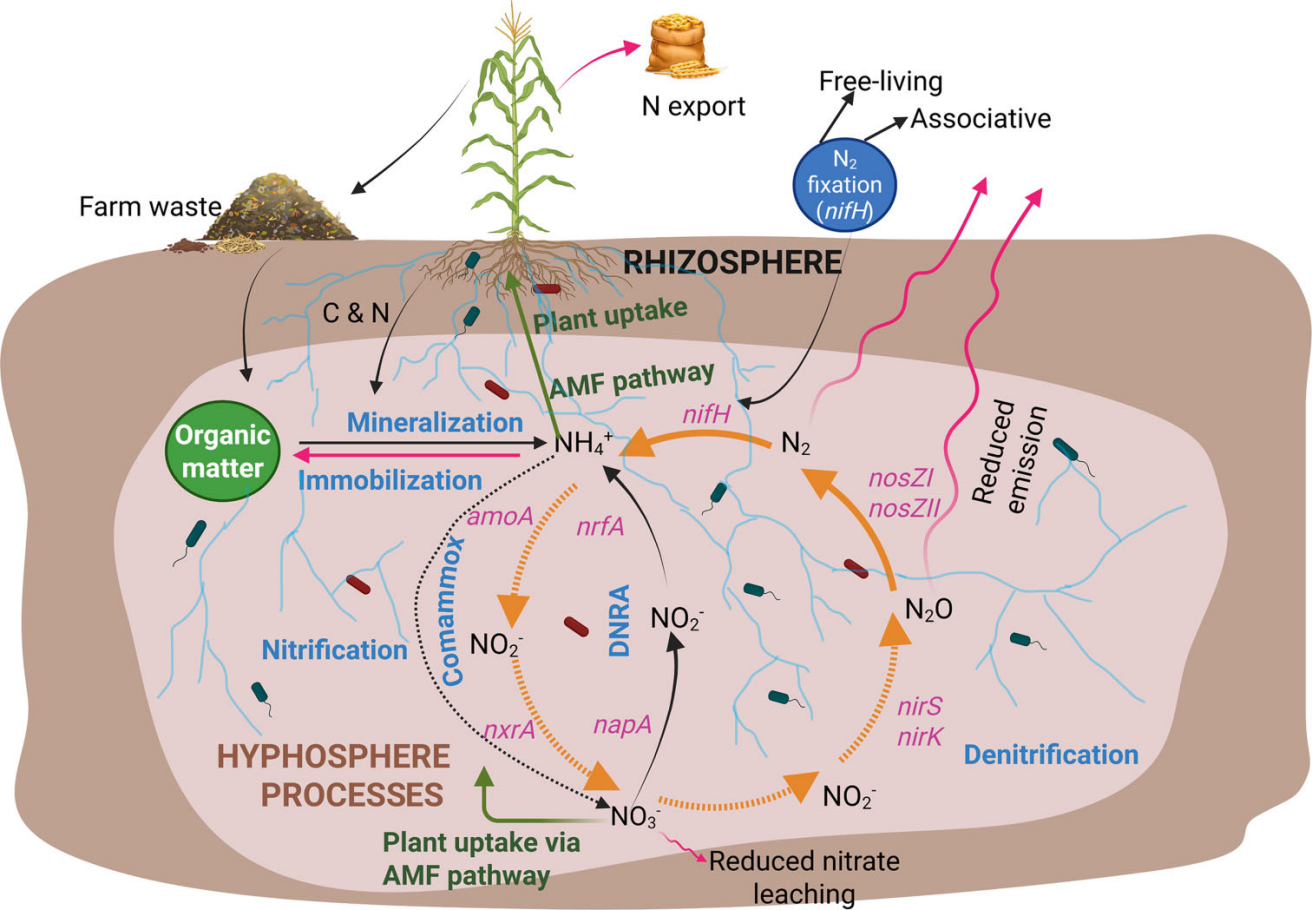
Conceptual model illustrating how AMF-driven hyphosphere processes can support more circular N management in agroecosystems. Illustration of how AMF-plant-microbe interactions may contribute to a semi-closed, circular N system. Plant-derived carbon fuels hyphosphere microbial networks that depolymerize organic N, retain mineral N through DNRA and microbial immobilization, and supply ammonium and amino-N to AMF hyphae to cover their own metabolic needs and for transfer to host plants. Together, these hyphosphere processes enhance soil internal N recycling and reduce environmentally harmful losses. Within the hyphosphere, AMF hyphae influence microbial guilds responsible for nitrification (carrying genes like *amoA* or *nxrA*), DNRA (*nrfA, napA*), denitrification (*nirK, nirS, nosZ-I, nosZ-II*), and biological N₂ fixation (*nifH*), thereby shaping the direction and magnitude of N fluxes. Dotted arrows denote downregulated processes, whereas solid arrows indicate processes that are stimulated within AMF-dominated zones. For example, AMF hyphae suppress nitrification, reduce nitrate leaching, and enhance *nosZ*-type reductase activity, promoting more complete denitrification and lowering N₂O emissions. Rapid hyphal uptake of ammonium further limits nitrate accumulation, contributing to reduced leaching. Although N export via harvest and some leaching or gaseous losses are unavoidable, synchronizing recycled N inputs (residues, manure, compost, digestate) with periods of high root and AMF hyphal activity can improve internal N cycling efficiency and help decrease reliance on synthetic N fertilizers. Created with BioRender.com.

We developed a conceptual model highlighting how AMF can enhance internal ecosystem N recycling, reduce N losses, and support more circular N management despite unavoidable N export through harvested crop biomass (Fig. 3). This model integrates mineralization, immobilization, nitrification, DNRA, denitrification, biological N₂ fixation, and plant uptake pathways within the rhizosphere-hyphosphere continuum, highlighting how AMF influence both N retention and N transformation processes. At the entry point of recycled inputs (e.g. residues, compost, manure, digestate), organic N is mineralized to ammonium (NH₄⁺), which represents a key branching node in the system. As illustrated in Fig. 3, NH₄⁺ may be (i) directly assimilated by plants directly or indirectly via the AMF pathway, (ii) immobilized into microbial biomass, (iii) oxidized through nitrification to nitrate (NO₃⁻), or (iv) the nitrate reduced via DNRA back to NH₄⁺. By extending beyond root depletion zones, AMF hyphae increase the spatial capture of NH₄⁺ and NO₃⁻, effectively intercepting mineral N before it is lost through leaching or gaseous emissions.

This model further highlights how AMF may indirectly influence the partitioning of NO₃⁻ between denitrification and DNRA pathways within the hyphosphere. As discussed above, enhanced C availability near AMF hyphae may favour nitrate ammonification over denitrification, thereby conserving N in ammonium form rather than releasing/losing it as N₂O or N₂. In parallel, stimulation of *nosZ*-containing denitrifiers may enhance the reduction of N₂O to N₂, lowering GHG emissions. Although these shifts remain context dependent, they illustrate how AMF-associated microbial processes could modulate both N retention and emission pathways. Biological N₂ fixation represents an additional input pathway shown in Fig. 3. Free-living and associative diazotrophs in the rhizosphere and hyphosphere may benefit from AMF-mediated carbon fluxes, reinforcing N inputs under low-N conditions. While unlikely to fully compensate for N export in harvested biomass, such inputs may partially offset deficits in reduced-input systems. Importantly, the model also acknowledges unavoidable N export through harvested grain or biomass. Therefore, AMF-driven recycling alone cannot sustain long-term system balance without returning ON sources to the soil. Circular N management thus requires coupling AMF-mediated interception and recycling with external recycled inputs that replenish exported N. The effectiveness of this coupling will depend on synchronizing organic amendments with periods of active hyphal growth and high microbial turnover, when mineralization, immobilization, and fungal uptake are most effective (Yang et al. 2024).

## Conclusion and future research directions

This article synthesizes information on AMF and their role in soil N cycling processes linked to redistribution of plant-derived C into the hyphosphere. Together, all the collective evidence suggests that AMF can influence N retention, redistribution, and loss pathways in soils. However, significant uncertainties remain regarding spatial mechanisms, species-specific effects, and field-scale relevance of AMF functioning. Addressing these gaps requires a coordinated application of complementary methodological approaches and emerging technologies.

Compartmental microcosm systems, which include roots and hyphae combined with ^15^ N labeling, allow for separation of fungal, plant, and microbial N uptake pathways. These systems are essential for determining whether AMF act as net facilitators or competitors for mineral N. At the ecosystem scale, ^15^ N pool dilution and isotope mass-balance approaches can facilitate the partitioning of nitrification, DNRA, and denitrification pathways under field conditions. Such classical methods are indispensable for estimating N budgets and quantify various N cycling processes.

In addition, high-resolution imaging platforms, such as NanoSIMS (Nanoscale secondary ion mass spectrometry), can visualize isotope incorporation into hyphae and associated microbial cells in the hyphosphere (Kaiser et al. 2015, Mayerhofer et al. 2021). This technology can provide direct evidence of the spatial coupling between C release and N transformations. While these micro-scale spatial measurements are critical for understanding hyphosphere mechanisms, they often require destructive and temporally static approaches. Furthermore, metagenomics and meta-transcriptomics coupled with stable isotope probing (SIP) analyses can detect microbial guilds active in N cycling processes within the hyphosphere (Dumont and Hernández 2019, Nuccio et al. 2022). Additionally, coupling functional gene expressions (e.g. *amoA, nrfA, nirS, nosZ, nifH*) with isotope incorporation will deepen our understanding of whether AMF selectively stimulate microbial guilds that conserve N as opposed to those that promote its loss. These approaches directly link microbial identity to the functions responsible for N turnover in the AMF hyphosphere.

Moreover, the classical trait-based measurements of AMF are essential for understanding how these traits influence N acquisition. Key features include hyphal length density, AMF colonization structures within roots, extraradical biomass, growth dynamics, and nutrient stoichiometry (Souza 2015, Ferrol and Lanfranco 2020). Recent studies have begun to focus on the traits to enhance understanding the functionality of AMF (Antunes et al. 2025, Chaudhary et al. 2025). By applying these approaches alongside genomics and transcriptomics on different AMF isolates, we can explore whether variations in N pools exploitation are largely driven by intrinsic properties of the fungi or by differences in genes related to expression of N transporters or other metabolic pathways. Additionally, studies that utilize synthetic AMF communities or multiple isolates of the same species could help clarify how intra-specific variation contributes to functional diversity in N acquisition. Finally, connecting the variation in AMF traits with an analysis of hyphosphere microbial communities will be vital for determining whether functional diversity is mainly driven by fungal physiology, interactions with associated microbes, or a combination of both.

Overall, the combination of the above approaches are essential for resolving AMF-mediated N cycling processes and for developing mechanistic, data-driven network models. By integrating these data streams with stoichiometric and transport models, we can gain insight into the trade-offs in N acquisition under different environmental conditions. Long-term field trials that monitor soil ON and mineral N dynamics, GHG fluxes, and hyphal abundance through sensor networks will be crucial for incorporating AMF-driven N transformations into soil nutrient cycling and for developing sustainable nutrient management strategies in agricultural and natural ecosystems.
